# Health-related quality of life among inpatients suffering from Traumatic brain injury in an urban setting in Vietnam

**DOI:** 10.1371/journal.pone.0308372

**Published:** 2024-08-06

**Authors:** Hai Minh Vu, Nam Nhu Duong, Cuong Duy Nguyen, Linh Gia Vu, Hien Thu Nguyen, Tung Hoang Tran

**Affiliations:** 1 Department of Trauma, Thai Binh University of Medicine and Pharmacy, Thai Binh, Vietnam; 2 Department of Intensive Care Unit, Thai Binh University of Medicine and Pharmacy, Thai Binh, Vietnam; 3 School for Preventive Medicine and Public Health, Hanoi Medical University, Hanoi, Vietnam; 4 Institute for Global Health Innovations, Duy Tan University, Da Nang, Viet Nam; 5 Faculty of Medicine, Duy Tan University, Da Nang, Vietnam; 6 Institute of Orthopaedic and Trauma Surgery, Vietnam–Germany Hospital, Hanoi, Vietnam; Iran University of Medical Sciences, ISLAMIC REPUBLIC OF IRAN

## Abstract

**Background:**

Traumatic brain injury (TBI) is a leading cause of mortality and morbidity globally. This study aimed to measure the health-related quality of life (HRQOL) of TBI patients in Vietnam and evaluate the associated factors.

**Methods:**

A longitudinal study was conducted in Thai Binh, Vietnam, from February to September 2020. The EuroQol-5 Dimensions-5 Levels (EQ-5D-5L) and EQ-Visual Analog Scale (EQ-VAS) were used to measure HRQOL. Socio-demographic characteristics, including age, gender, education level, employment status, marital status, and income, as well as clinical characteristics such as injury severity, cause of injury, comorbidities, and functional status, were collected through patient interviews and medical records. Multivariate Tobit regression analysis was performed to identify variables associated with HRQOL.

**Results:**

The study included 212 TBI patients. The mean scores of the VAS and EQ-5D-5L scales were 82.4/100 (SD = 14.49) and 0.9/1.0 (SD = 0.2), respectively, indicating a relatively high HRQOL. However, certain factors significantly impacted HRQOL. Unemployed patients and those with depression or higher injury severity, as measured by the Injury Severity Score (ISS), had notably lower HRQOL scores. Poor sleep quality and severe functional impairments also adversely affected HRQOL, whereas patients discharged for a longer period had slightly better HRQOL scores.

**Conclusion:**

This study highlights that while HRQOL is relatively high among TBI patients, mental health disorders, injury severity, and functional impairments are key factors negatively impacting their quality of life. High HRQOL was defined by mean scores on the VAS and EQ-5D-5L scales, with higher scores indicating better quality of life. Home-based interventions addressing these issues could improve HRQOL for TBI patients.

## 1. Introduction

Traumatic brain injury (TBI) is a leading cause of death and disability in both developed and developing countries, with an increasing incidence among older adults. The global prevalence of TBI varies, with reported incidences as high as 500 per 100,000 people [1]. Between 1990 and 2013, TBI resulted in a loss of 247.6 million disability-adjusted life years worldwide [2,3]. The significant health and socio-economic impacts predominantly affect the young working population, while the diverse and often undiagnosed psychiatric consequences hinder patients’ ability to return to their previous jobs.

There has been growing awareness and attention towards health-related quality of life (HRQOL) as an important outcome measure following trauma, particularly TBI [4,5]. Research has shown that even a single episode of TBI can lead to disability and reduced HRQOL compared to the general population [4,6]. Numerous studies indicate that trauma, including TBI, is associated with a decline in HRQOL, often accompanied by prolonged pain and anxiety [7,8]. However, findings on the impact of injury severity on HRQOL vary. Some studies report that severe TBI leads to deteriorated HRQOL [9,10], while others find no significant association [11,12] or suggest a complex and possibly contradictory relationship [13,14]. TBI combined with polytrauma has been identified as a factor contributing to diminished HRQOL, with those experiencing both TBI and polytrauma showing more significant impairments than those without TBI [15,16]. Additional predictors of HRQOL in TBI patients may include gender, employment and leisure activity engagement, and emotional support availability [17]. However, there is limited research comparing HRQOL among trauma patients, specifically focusing on TBI.

Despite the availability of several TBI-specific tools for assessing health-related quality of life (HRQOL), such as the Quality of Life After Brain Injury Overall Scale [18] and the Traumatic Brain Injury Caregiver Quality of Life [19], the EuroQol-5 Dimensions-5 Levels (EQ-5D-5L) questionnaire was used for this study. The EQ-5D-5L is a widely recognized, generic, and preference-based instrument that allows for broad comparisons across different populations and conditions. This tool is not only well-established in many countries but also has normative data available, which facilitates benchmarking and comparative analysis. Additionally, the EQ-5D-5L has been extensively validated across various health conditions, including cancer and metabolic disorders [20], making it a robust and versatile instrument for assessing HRQOL. Previous studies have effectively used the EQ-5D-5L to compare HRQOL in individuals with post-concussion syndrome (PCS) after TBI and those without, highlighting its sensitivity in detecting differences in HRQOL [21]. Another study found a high EQ-5D-5L index among recovered TBI patients, correlated with the Glasgow Coma Scale score, Glasgow Outcome Scale, pupillary light reflex, and surgery [22]. Therefore, the use of the EQ-5D-5L in our study allows for comprehensive assessment and comparison of HRQOL in TBI patients within the broader context of general health conditions.

TBI is a significant issue in Vietnam [23]. A 2016 report indicated that there were over 605,000 TBI cases, with a prevalence rate of 644 per 100,000 people [23]. TBI poses a substantial economic burden and can be devastating for patients’ families [24]. Therefore, more evidence on TBI treatment outcomes, especially HRQOL, is needed to develop appropriate treatment plans for TBI patients. This study aims to measure the HRQOL of TBI patients in Vietnam and evaluate asociations between HRQOL and different factors including socio-demographic characteristics, clinical characteristics, mental health status, sleep quality, and social support.

## 2. Materials and methods

### 2.1. Study design and participants

A longitudinal study was conducted in Thai Binh, Vietnam, from February 1 to September 31, 2020. Participants had to meet the following inclusion criteria: 1) be over 18 years old; 2) currently reside in Vietnam; and 3) have a diagnosis of traumatic brain injury (TBI). Participants under 18 were not included to ensure that all participants could provide informed consent and fully understand and respond to the questionnaire independently. Additionally, the clinical presentation and treatment outcomes of TBI can differ significantly between adults and children, necessitating separate studies for these age groups.

Participants with severe illnesses, significant cognitive impairments, or those unable to respond to the data collectors’ questions were excluded from the study. Patients were recruited from the Department of Neurosurgery and Spine at Thai Binh Provincial General Hospital. Recruitment was based on convenience from among TBI patients admitted during the study period. Initially, socio-demographic and clinical characteristics were examined. After at least three months post-hospital discharge, the research team conducted a follow-up assessment. A total of 220 patients were invited to participate, and 212 completed the follow-up interview. The study protocol was approved by the Institutional Review Board of Thai Binh University of Medicine and Pharmacy (Code: 1183/HDDD).

### 2.2. Measure and instruments

A structured questionnaire was administered, covering six primary sections: 1) Socio-demographic factors; 2) Sleep quality, health status, and use of healthcare services; 3) Activities of daily living; 4) Social support (measured using the MSPSS scale); 5) Traumatic brain injury characteristics and severity; and 6) HRQOL. Telephone interviews, averaging about 20 minutes each, were conducted for their cost-effectiveness and efficiency in managing patient populations. Post-discharge interviews were scheduled within one to eight months. Participation required consent from the individuals. The investigators received training in administering questionnaire interviews. A preliminary survey was conducted with a diverse sample of 20 individuals of different ages, genders, and parental characteristics to ensure the cross-cultural validity of the Vietnamese-translated questionnaire. These 20 participants were not included in the primary survey. Data collection began after thoroughly testing the online survey system to ensure the platform’s reliability, the accuracy of survey content, and the absence of technical issues. The semi-structured questionnaire was successfully completed, covering the main sections.

#### 2.2.1. Outcome variables. Health-related Quality of Life (HRQOL)

To assess the HRQOL of participants, we used the Visual Analogue Scale (VAS) and the EQ-5D-5L. These scales evaluated HRQOL on the day of the interview [25]. The VAS score ranges from 0 to 100, with higher scores indicating better HRQOL. The EQ-5D-5L assesses five domains: Mobility, Self-care, Usual Activities, Pain/Discomfort, and Anxiety/Depression. Each domain is scored from 1 (no problems) to 5 (extreme problems), resulting in 3,125 possible health states, from the worst health (55555) to the best (11111) [26]. Each health state is given a "utility" score, which can range from -0.5112 to 1 in the Vietnamese version used in this study [27]. Higher scores indicate better HRQOL. The Cronbach’s alpha coefficient for the EQ-5D-5L was 0.91, indicating strong internal consistency. The validity and reliability of the Vietnamese-translated version of the EQ-5D-5L have been previously evaluated and confirmed [28].

#### 2.2.2. Covariates. Socio-economic and Clinical Characteristics

Participants answered questions about gender (male/female), job (student/officials/freelance/others), education level (high school or lower/tertiary or higher), marital status (single/married), household economic status (low/average/high), and average monthly income. They also reported on the cause and severity of their TBI and any other injuries combined with TBI.

**Traumatic Brain Injury Characteristics:** Participants self-reported the cause of their TBI, the severity of the injury, and whether they had other injuries along with the TBI. The Injury Severity Score (ISS) was used to assess overall severity, covering six domains: Head/Neck, Face, Chest, Abdomen, Extremities, and External. Scores range from 0 to 75, with an AIS score of 6 automatically resulting in an ISS of 75, indicating an untreatable injury [29].

**Health Risk Behavior:** We assessed cigarette smoking and alcohol consumption. Participants reported if they were current smokers or not during the study period. Alcohol consumption was evaluated using the AUDIT-C scale, consisting of three questions with a total score of 12 points. A score of 4 or more for men and 3 or more for women indicated a positive AUDIT-C classification. Participants also reported binge drinking, defined as consuming more than six alcoholic beverages on one occasion [30].

**Health Status and Healthcare Services:** Participants provided information about their sleep quality and injury severity. We used the Pittsburgh Sleep Quality Index (PSQI) to measure sleep quality, with scores ranging from 0 to 21. A score of 5 or more indicates poor sleep quality. Participants also reported their duration of hospitalization, comorbidities, initial medical assistance, and timing of the interview after discharge to understand their use of healthcare services [31].

**Activity of Daily Living (ADL):** The ADL scale measured self-care and personal hygiene activities across seven domains: bathing, dressing, toileting, maintaining continence, grooming, feeding, and transferring. Scores range from 0 to 6, with 6 indicating full functionality, 4 indicating moderate impairment, and 2 or below indicating significant impairment [32].

**Social Support:** We used the Multidimensional Scale of Perceived Social Support (MSPSS) to measure social support from family, friends, and significant others. Higher scores indicate greater perceived social support [33].

### 2.3. Statistical analysis

Data analysis was conducted using STATA version 16 (Stata Corp. LP, College Station, USA). We addressed missing data using the Listwise Deletion method before analysis. Continuous variables were presented as mean and standard deviation (SD), while categorical variables were expressed as frequencies and percentages. The Chi-squared and Kruskal-Wallis tests were used to evaluate differences between male and female participants. Potential covariates for the comprehensive models included individual characteristics, sleep quality, health status, healthcare service utilization, daily functioning abilities, social support (measured by the MSPSS scale), and the characteristics and severity of traumatic brain injury. A multivariate Tobit regression analysis was performed to identify variables associated with HRQOL based on the VAS and EQ-5D-5L scales. Stepwise forward selection strategies were used to generate condensed models, including variables with a threshold of p<0.2. Statistical significance was set at p<0.05.

## 3. Results

Table 1 outlines the socio-economic data of the study participants. Out of 212 participants, 67.5% were male. The average age was 47.1 years (SD = 17.6). Most respondents (84.9%) lived in rural areas, with a significant difference between genders. The largest occupational groups were workers/farmers (36.8%) and freelance workers (33.5%). Approximately 70% were married or partnered. The average monthly income was $469.4 (SD = 227.8). Hazardous alcohol use was reported by 32.1% of participants, and 76.9% were current smokers. There was a significant difference in alcohol and tobacco use between men and women (p < 0.01).

**Table 1:** Individual characteristics of participants (n = 212).

Characteristics	Gender				Total		p-value
	Male		Female				
	n	%	n	%	n	%	
**Total**	143	67.5	69	32.5	212	100	** **
**Occupation**							
Freelance work	46	32.2	25	36.2	71	33.5	0.55
Workers/Farmers	26	18.2	13	18.8	39	18.4	
Other jobs	63	44.1	30	43.5	93	43.9	
Unemployment	8	5.6	1	1.5	9	4.3	
**Education**							
High school or lower	115	80.4	59	85.5	174	82.1	0.66
Intermediate/ College/ Vocational education	16	11.2	6	8.7	22	10.4	
Tertiary or upper	12	8.4	4	5.8	16	7.6	
**Current location**							
Urban	15	10.5	17	24.6	32	15.1	<0.01
Rural	128	89.5	52	75.4	180	84.9	
**Marital**							
Single	35	24.5	14	20.3	49	23.1	0.60
Married/Partnered	100	69.9	49	71.0	149	70.3	
Divorced/ Separated/ Widowed	8	5.6	6	8.7	14	6.6	
**Hazardous drinking**	66	46.2	2	2.9	68	32.1	<0.01
**Current smoker**	98	68.5	65	94.2	163	76.9	<0.01
**Comorbidities**							
0	109	76.2	48	69.6	157	74.1	0.39
1	24	16.8	17	24.6	41	19.3	
≥2	10	7.0	4	5.8	14	6.6	
**Caregiver**							
Parents/ Yokefellow	109	76.2	46	66.7	155	73.1	0.14
Offsprings	45	31.5	29	42.0	74	34.9	0.13
Grandchildren/ Helpers	12	8.4	8	11.6	20	9.4	0.46
	**Mean**	**SD**	**Mean**	**SD**	**Mean**	**SD**	**p-value**
**Age**	46.8	18.3	47.6	16.1	47.1	17.6	0.70
**Monthly income (USD)**	479.7	249.3	448.2	174.7	469.4	227.8	0.90

Table 2 shows the TBI characteristics and quality of life of participants. The mean quality of sleep score was 8.8 (SD = 3.0). The average hospital stay was 10.68 days (SD = 5.25). Traffic accidents were the leading cause of TBI (65.6%), followed by falls (20.3%), with a statistically significant difference between males and females (p = 0.03). Mild traumatic brain injury was the most common, affecting 93.9% of patients. Additionally, 62.2% of patients had TBI along with other injuries. The mean Injury Severity Score (ISS) was 9.7 out of 75 (SD = 6.5), with a significant difference between male and female patients (p = 0.01). The mean scores for the VAS and EQ-5D-5L scales were 82.4 out of 100 (SD = 14.49) and 0.9 out of 1.0 (SD = 0.2), respectively. The mean social support scores were 5.4 from Family, 4.7 from Friends, and 5.9 from Others.

**Table 2:** Characteristics of traumatic brain injury and quality of life of participants.

Characteristics	Gender				Total		p-value
	Male		Female				
	n	%	n	%	n	%	
**Cause of injury**							
Fall	34	23.8	9	13.0	43	20.3	0.03
Traffic accidents	94	65.7	45	65.2	139	65.6	
Others	15	10.5	15	21.7	30	14.2	
**Severity of injury**							
Mild TBI	131	91.6	68	98.6	199	93.9	<0.05
Moderate/Severe TBI	12	8.4	1	1.5	13	6.1	
**Traumatic brain injury combined with other injuries**	92	65.7	38	55.1	130	62.2	0.14
**PHQ9 Index**							
Minimal or none	117	81.2	49	71.0	166	78.3	0.07
Mild/Moderate/Moderately severe	26	18.2	20	29.0	46	21.7	
**Activities of Daily Living**							
Full function	105	73.4	56	81.2	161	75.9	0.42
Moderate impairment	29	20.3	9	13.0	38	17.9	
Severe functional impairment	9	6.3	4	5.8	13	6.1	
**Quality of Sleep**							
Normal	55	38.5	22	31.9	77	36.3	0.35
Poor Sleep	88	61.5	47	68.1	135	63.7	
	**Mean**	**SD**	**Mean**	**SD**	**Mean**	**SD**	**p-value**
**The number of days hospitalized**	10.8	5.4	10.4	4.9	10.7	5.2	0.74
**Interview time after discharge**	3.6	1.9	3.4	1.7	3.5	1.8	0.38
**Injury Severity Score (0–75)**	10.5	6.8	8.1	5.4	9.7	6.5	0.01
**VAS score (0–100)**	83.2	13.8	80.7	15.7	82.4	14.5	0.24
**EQ-5D-5L Utility**	0.8	0.2	0.9	0.2	0.9	0.2	0.99
**Measurement of perceived social support**							
Family group (range 0 to 7)	5.4	0.8	5.3	0.9	5.4	0.8	0.93
Friend group (range 0 to 7)	4.7	1.0	4.7	0.9	4.7	0.9	0.68
Other group (range 0 to 7)	5.1	1.0	4.9	1.2	5.0	1.0	0.39

Table 3 provides evidence on factors associated with participants’ HRQOL as assessed using the EQ-5D-5L and VAS scales. Unemployed participants had significantly lower VAS scores compared to freelance workers (Coef. = -9.63; 95% CI = -19.00, -0.26). Patients with symptoms of depression had significantly lower scores on both the EQ-5D-5L (Coef. = -0.14; 95% CI = -0.21, -0.06) and the VAS (Coef. = -6.97; 95% CI = -12.47, -1.46). A higher Injury Severity Score (ISS) was associated with decreases in both EQ-5D-5L (Coef. = -0.01; 95% CI = -0.01, 0.00) and VAS scores (Coef. = -0.36; 95% CI = -0.67, -0.05).

**Table 3:** Multivariate Tobit regression for identifying factors associated with HRQOL of patients following TBI.

FACTORS	EQ-5D-5L		VAS	
	Coef	95%CI	Coef	95%CI
**SOCIO-ECONOMIC STATUS**				
**Age (per age)**	0.02	-0.05; 0.08	-0.15*	-0.32; 0.02
**Current location (vs. Urban -Ref)**				
Rural	0.03	-0.04; 0.10	0.53	-4.27; 5.33
**Education (vs High school or lower -Ref)**				
Intermediate/ College/ Vocational education	-0.01	-0.09; 0.07	1.22	-4.52; 6.96
Tertiary or upper	-0.00	-0.12; 0.12	-3.50	-11.13; 4.12
**Marital (vs Single -Ref)**				
Married/Partnered	-0.02	-0.10; 0.07	2.03	-3.71; 7.76
Divorced/ Separated/ Widowed	0.01	-0.14; 0.16	11.42**	1.02; 21.82
**Job (vs Freelancer -Ref)**				
Workers/Farmers	-0.02	-0.11; 0.06	3.92	-1.85; 9.69
Other jobs	0.01	-0.04; 0.07	2.79	-1.07; 6.64
Unemployment	-0.05	-0.18; 0.08	-9.63**	-19.00; -0.26
**Current smoking (vs No -Ref)**				
Yes	-0.09**	-0.16; -0.02	-1.13	-5.62; 3.37
**Hazardous (vs No -Ref)**				
Yes	0.01	-0.06; 0.07	1.13	-3.29; 5.55
**Health Insurance (vs. No -Ref)**				
Yes	0.03	-0.05; 0.11	0.53	-4.81; 5.87
**CLINICAL CHARACTERISTICS**				
**Comorbidities (vs 0 -Ref)**				
1	0.02	-0.05; 0.09	-0.79	-5.34; 3.76
≥2	-0.06	-0.16; 0.05	-2.97	-10.34; 4.40
**Cause of injury (vs. Fall -Ref)**				
Traffic accidents	0.04	-0.03; 0.10	2.28	-2.35; 6.92
Other	0.05	-0.04; 0.14	4.74	-1.32; 10.81
**Traumatic brain injury combined with other injuries (vs No -Ref)**				
Yes	0.02	-0.03; 0.08	0.57	-3.42; 4.56
**The severity of injury (vs. Mild TBI -Ref)**				
Moderate/Severe TBI	0.08	-0.03; 0.19	-0.04	-7.67; 7.59
**PHQ9 Depression (vs Minimal or none)**				
Mild/Moderate/Moderately severe	-0.14***	-0.21; -0.06	-6.97**	-12.47; -1.46
**Activities of Daily Living (vs. Full function -Ref)**				
Moderate impairment	-0.05	-0.13; 0.02	-4.16	-9.25; 0.93
Severe functional impairment	-0.34***	-0.47; -0.22	-17.77***	-26.70; -8.83
**Quality of Sleep (vs. Normal sleep -Ref)**				
Poor Sleep	-0.06**	-0.12; -0.00	-3.44*	-7.31; 0.43
**Injury Severity Score (0–75)**	-0.01***	-0.01; -0.00	-0.36**	-0.67; -0.05
**Interview time after discharge (per month)**	0.02**	0.00; 0.03	0.49	-0.63; 1.60
**The number of days hospitalized (per day)**	0.00*	-0.00; 0.01	0.20*	-0.00; 0.40
**SOCIAL SUPPORT**				
**Measurement of perceived social support**				
Family group range (per score)	-0.00	-0.05; 0.05	-2.19	-5.50; 1.11
Friend group range (per score)	-0.03	-0.07; 0.01	2.65*	-0.14; 5.44
Other group range (per score)	0.02	-0.02; 0.06	0.33	-2.39; 3.04

*p<0.1

**p<0.05

***p<0.01.

Patients with poor sleep or severe functional impairment had lower HRQOL scores compared to those with normal sleep (Coef. = -0.06; 95% CI = -0.12, 0.00 for EQ-5D-5L) or full function (Coef. = -0.34; 95% CI = -0.47, -0.22 for EQ-5D-5L and Coef. = -17.77; 95% CI = -26.70, -8.83 for VAS). Longer duration since hospital discharge was associated with higher EQ-5D-5L scores (Coef. = 0.02; 95% CI = 0.00, 0.03). Non-smokers had better quality of life compared to smokers (Coef. = -0.09; 95% CI = -0.16, -0.02).

## 4. Discussion

This study represents the first longitudinal cohort investigation of Vietnamese TBI patients to examine their HRQOL after hospital discharge using the EQ-5D-5L instrument. HRQOL is a health outcome assessed from patients’ viewpoints, aligning with holistic medicine principles [34]. This study showed high HRQOL among patients following TBI treatment. Various demographic, clinical, and social characteristics were associated with HRQOL, which could inform further home-based interventions to improve HRQOL.

The findings align with previous studies demonstrating diminished HRQOL among individuals with TBI compared to the general population and those without acute or chronic health conditions [28,35]. Our results showed slightly lower HRQOL compared to a prior study in Thailand, where the mean EQ-5D-5L index was 0.91558 (SD = 1.09639) and the mean EQ-VAS was 94.08 (SD = 9.71) [22]. In a Norwegian study, most participants with mild TBI reported lower HRQOL in physical health, mental health, social function, or role emotion [36]. An American mild-to-moderate TBI cohort also showed a notable decline in all HRQOL domains compared to a control group without injury [37]. However, the HRQOL of TBI patients was higher than that of patients with other chronic conditions such as diabetes (0.8) [38], skin diseases (0.73) [39], or respiratory diseases (0.66) [40], suggesting the effectiveness of current treatment methods in TBI recovery. This highlights the need to address factors contributing to HRQOL decline in TBI patients.

As expected, the analysis revealed a significant relationship between trauma severity and diminished HRQOL. Previous studies have reported similar findings, although not consistently [5,13]. Variations in data collection time, HRQOL assessment instruments, trauma severity, and patients’ cultural backgrounds may explain the divergent outcomes. This study confirmed the impact of TBI on HRQOL, emphasizing the importance of including HRQOL assessments in prognostic evaluations after TBI.

Multivariate regression analysis showed that, after adjusting for socio-demographic characteristics, patients with poor sleep quality, mental health disorders, and limited daily functions were more likely to experience compromised HRQOL following a TBI [41]. Depression and psychological distress frequently occur after severe TBI and are strongly associated with HRQOL [42]. A prior study found that patients exhibited diminished cognitive abilities, as evidenced by lower neuropsychological performance 12 months after injury [43]. Additionally, TBI patients showed a higher prevalence of depression compared to the general population [44].

Poor sleep quality is also common among TBI patients [45,46], with disturbances such as increased time to fall asleep, frequent night waking, and reduced overall sleep duration [47]. These disruptions often persist and are linked to prolonged recovery, lower quality of life, decreased job performance, and potential detriments to mental and physical well-being. These findings highlight the need to enhance rehabilitation interventions’ precision and specificity, particularly focusing on cognitive and psychological outcomes [48]. Providing personalized care throughout the rehabilitative journey requires collaborative efforts from healthcare professionals, family members, and patients.

There are several limitations to acknowledge. First, the cross-sectional study design limits causal conclusions. Recall bias and social desirability may have affected response accuracy, resulting in data underestimation or overestimation. The findings may not be generalizable to the Vietnamese population due to convenience sampling. To address these limitations, future studies should employ longitudinal designs to better establish causality, use random sampling to enhance generalizability, and incorporate a mix of data collection methods, such as face-to-face interviews and anonymous surveys, to reduce social desirability bias.

## 5. Conclusion

This study highlights the high HRQOL of patients after TBI. However, it also reveals that mental health disorders, injury severity, and limited daily functions significantly impair HRQOL. To improve patients’ HRQOL, home-based interventions should focus on addressing these issues. Practical strategies may include mental health support, rehabilitation programs tailored to injury severity, and assistance with daily activities. Implementing these targeted interventions can lead to better long-term outcomes for TBI patients.

## Abbreviations

HRQOL: Health-related quality of life
PCS: post-concussion syndrome
TBI: Traumatic brain injury
